# Abnormal Motor Phenotype at Adult Stages in Mice Lacking Type 2 Deiodinase

**DOI:** 10.1371/journal.pone.0103857

**Published:** 2014-08-01

**Authors:** Soledad Bárez-López, Daniel Bosch-García, David Gómez-Andrés, Irene Pulido-Valdeolivas, Ana Montero-Pedrazuela, Maria Jesus Obregon, Ana Guadaño-Ferraz

**Affiliations:** 1 Department of Endocrine and Nervous System Pathophysiology, Instituto de Investigaciones Biomédicas “Alberto Sols”, Consejo Superior de Investigaciones Científicas (CSIC)-Universidad Autónoma de Madrid (UAM), Madrid, Spain; 2 Trastornos del Desarrollo y Maduración Neurológica, IdiPAZ, Hospital Universitario La Paz, Madrid, Spain; 3 Departamento de Anatomía, Histología y Neurociencia, Universidad Autónoma de Madrid (UAM), Madrid, Spain; University of Vienna, Max F. Perutz Laboratories, Austria

## Abstract

**Background:**

Thyroid hormones have a key role in both the developing and adult central nervous system and skeletal muscle. The thyroid gland produces mainly thyroxine (T4) but the intracellular concentrations of 3,5,3′-triiodothyronine (T3; the transcriptionally active hormone) in the central nervous system and skeletal muscle are modulated by the activity of type 2 deiodinase (D2). To date no neurological syndrome has been associated with mutations in the *DIO2* gene and previous studies in young and juvenile D2-knockout mice (D2KO) did not find gross neurological alterations, possibly due to compensatory mechanisms.

**Aim:**

This study aims to analyze the motor phenotype of 3-and-6-month-old D2KO mice to evaluate the role of D2 on the motor system at adult stages in which compensatory mechanisms could have failed.

**Results:**

Motor abilities were explored by validated tests. In the footprint test, D2KO showed an altered global gait pattern (mice walked slower, with shorter strides and with a hindlimb wider base of support than wild-type mice). No differences were detected in the balance beam test. However, a reduced latency to fall was found in the rotarod, coat-hanger and four limb hanging wire tests indicating impairment on coordination and prehensile reflex and a reduction of muscle strength. In histological analyses of cerebellum and skeletal muscle, D2KO mice did not present gross structural abnormalities. Thyroid hormones levels and deiodinases activities were also determined. In D2KO mice, despite euthyroid T3 and high T4 plasma levels, T3 levels were significantly reduced in cerebral cortex (48% reduction) and skeletal muscle (33% reduction), but not in the cerebellum where other deiodinase (type 1) is expressed.

**Conclusions:**

The motor alterations observed in D2KO mice indicate an important role for D2 in T3 availability to maintain motor function and muscle strength. Our results suggest a possible implication of D2 in motor disorders.

## Introduction

Thyroid hormones (TH, T3 or 3,5,3′-triiodothyronine and T4 or thyroxine) have an important role in most tissues including the developing and adult central nervous system (CNS) and skeletal muscle. The thyroid gland synthesizes mostly T4 although most actions of TH are mediated by T3, the transcriptionally active form, by its binding to specific nuclear receptors modulating gene expression patterns [1]. In spite of this, mechanisms regulating intracellular T3 concentrations and their functional implications are not entirely understood. Among all the main control points, deiodinases (type 1, 2 and 3 or D1, D2 and D3) are important selenoenzymes in the metabolism of TH, regulating local T3 and T4 availability [2], [3]. D1 and D2 catalyze the removal of the iodine atom in the 5′ position of T4 (outer ring deiodinases), generating the genomically active hormone T3. D3 catalyzes the removal of the iodine from the 5 position of T4 or T3 (inner ring deiodinase) producing transcriptionally inactive metabolites, as reverse T3 (3,3′,5′-triiodothyronine or rT3) from T4.

In the CNS D2 is the main deiodinase that generates T3. D2 is mostly expressed in astrocytes throughout the brain [4], and tanycytes controlling local T3 production in brain and hypothalamic-pituitary axis [4], [5]. Important differences exist in regional and temporal expression of D2 in the CNS [6]–[8]. Constitutively, D2 is highly expressed in the median eminence and moderately expressed in many other regions of the CNS, many of them involved in motor control [6]. D2 is also expressed in murine and human skeletal muscle [7], [9]. In this tissue D2 also provides a significant contribution to the intracellular T3 concentrations and increases in D2 activity occur in normal myogenesis during development, in muscle repair after injury, and in hypothyroidism [10]–[12].

To the date, no inactivating mutations in the *DIO2* gene in humans have been reported. However, *DIO2* gene polymorphisms have been associated with mental retardation and bipolar disorder [13]–[15] and insulin resistance [16], [17], unrevealing the importance of D2 in CNS and muscle.

The generation of a D2 knockout (D2KO) mouse [18] has highlighted the importance of the local regulation of T3 availability by D2 in several tissues. Studies in D2KO mice have demonstrated the implication of D2 in the regulation of the hypothalamic-pituitary-thyroid (HPT) axis [18], the thermogenesis in brown adipose tissue [19], the cochlear development [20], the development and repair of skeletal muscle [11], [12] and the optimization of bone strength [21].

Considering the role of D2 in modulating TH signaling during development and at adult stages [2], its expression in somatosensory and motor systems [6], the marked motor alterations in congenital hypothyroidism [22], and that motor function provides a good read-out of neurological function, D2KO mice were expected to show an evident abnormal motor phenotype with an early onset. However, only very mild neurological signs have been reported between 2.5 to 3 months of age in D2KO mice, probably due to important compensatory mechanisms. Several homeostatic processes have been proposed as possible compensatory mechanisms in the absence of D2 activity such as an increased T3 transport to brain parenchyma coming from serum and/or the cerebrospinal fluid, and putative genomic and non-genomic T4 actions in the brain [23].

In the present study we have aimed to better-define the neurological phenotype of D2KO mice in order to improve the understanding of the role of D2 in motor system physiology and pathophysiology. We have focused on the role of D2 in motor processes at adult stages, when a long-term failure of sustained compensatory mechanisms and/or cumulative damage could have occurred and diseases could appear. To accomplish this aim, we have further characterized the motor phenotype of D2KO mice and expanded the phenotypic analysis to 6-months-old mice.

Our results indicate that adult D2KO mice show an abnormal performance of several motor tasks suggesting that D2 plays a significant role in the development and/or function of the motor system. In addition, our studies indicate that the adult D2KO mouse could be a good model to deeply analyze the role of D2 in the CNS and muscle.

## Materials and Methods

### Ethics Statement

All experimental procedures were performed following the European Union Council guidelines (2010/63/EU) and Spanish regulations (RD 1201/2005 and RD 53/2013) for the use of laboratory animals in chronic experiments, and were approved by the Bioethics committee of our Institution (Consejo Superior de Investigaciones Científicas; approval numbers PN2007-116 and PN2010-55). All efforts were made to minimize suffering and are indicated below.

### Experimental Animals

D2KO mice and wild-type (WT) counterparts were initially provided by Dr. Galton [18] and a colony was established at Instituto de Investigaciones Biomédicas (Madrid, Spain). *Dio2* mutation was backcrossed for 3 further generations onto the C57BL/6 background. The number of animals used in each study is stated in the figures and results section. Mice were housed in plastic cages in an air-conditioned and light controlled room at 24±2°C and 60% ±5% humidity with automatic light/dark cycles of 12/12 h. Three and 6-months-old male WT and D2KO mice were used.

A decrease of body weight gain is a good biomarker of TH deficiencies in rodents. WT and D2KO mice’s body weight was monitored in 3, 4, 5, 6 and 12 month-old mice in at least 20 animals of each age and experimental condition. As previously reported in 2-months-old mice [18], no significant differences in body weight were observed between WT and D2KO mice in any of the studied ages (Table 1).

**Table 1:** Body weight in WT and D2KO mice at different ages.

Genotype	3 months	4 months	5 months	6 months	12 months
WT	27.4±0.6 (22)	29.3±0.7 (36)	31.1±0.4 (35)	32.0±0.7 (20)	36.6±1.1 (22)
D2KO	27.7±0.4^NS^ (25)	28.0±0.5^ NS^ (29)	30.6±0.5^ NS^ (27)	32.5±0.9^ NS^ (21)	35.7±1.6^ NS^ (20)

Data are means ± SD in grams. NS, not statistically significant as compared to WT control group. The number of animals is stated in parenthesis.

For histological analyses of the CB, mice were anesthetized by intraperitoneal injection of a mixture of ketamine and medetomidine (75 mg and 1 mg per kg of body weight, respectively) and transcardially perfused with 4% paraformaldehyde in 0.1 M PB. Brains were removed, post-fixed overnight in the same fixative solution, cryoprotected in 30% sucrose, and 25 µm free-floating sections were serially cut on a cryostat in the coronal plane.

For histological analysis of the skeletal muscle, intact *gastrocnemius* and *soleus* muscles were dissected from mice sacrificed by decapitation, post-fixed in 4% paraformaldehyde in 0.1 M phosphate buffer (PB) for 48 h, processed for paraffin embedding and sectioned at 5 µm in the transversal plane.

For biochemical analyses, mice were sacrificed by decapitation and blood was collected using heparinized tubes. Tissues (cerebellum [CB], cerebral cortex [CTX], and quadriceps muscle were extracted from every animal and rapidly frozen on dry ice and stored at −70°C until processed.

### Motor tasks assessment

#### Footprint test

**Figure 1:**
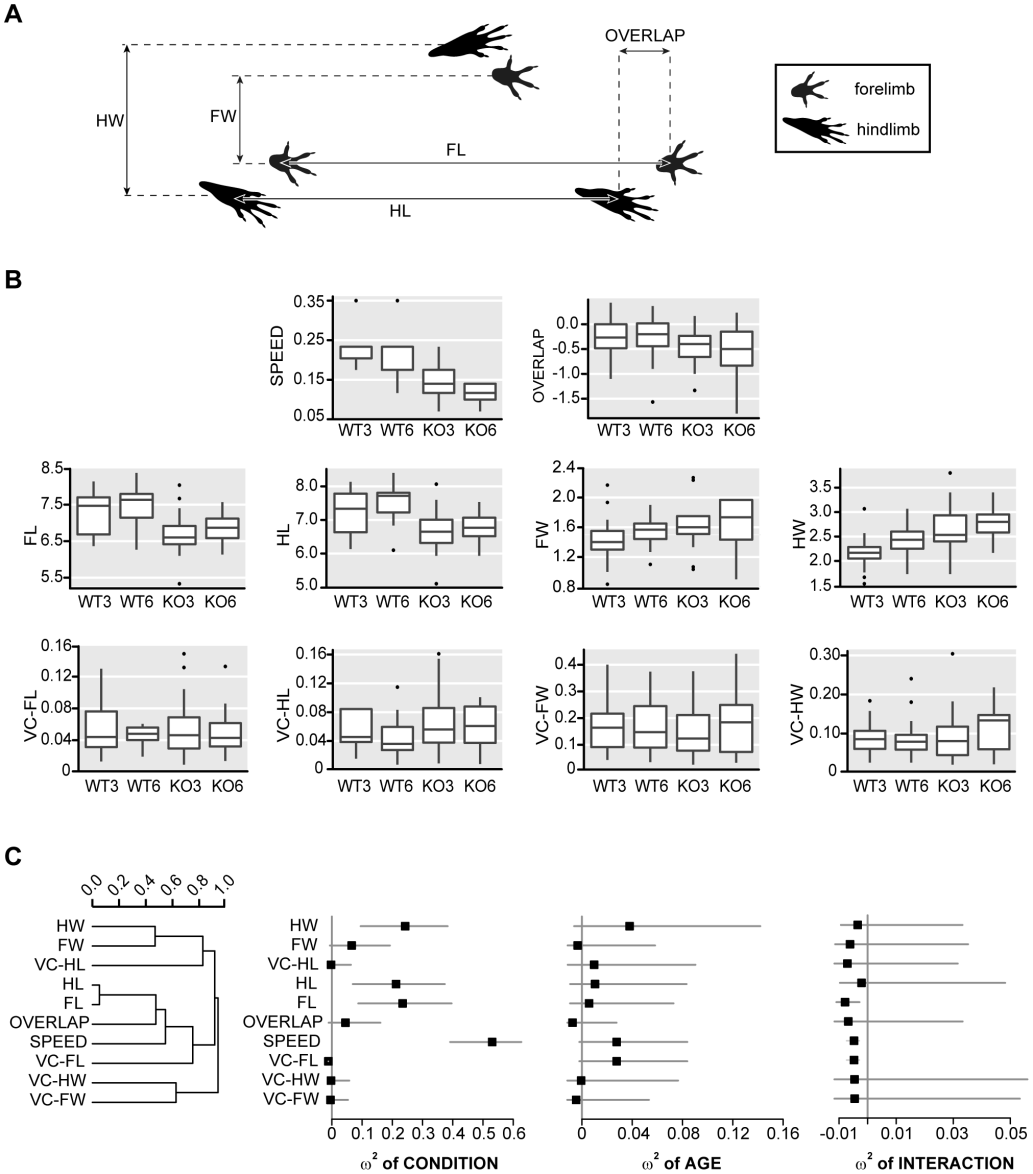
Alteration in footprint test. **A.** Schematic representation of variables measured in footprint test (FW, front base width; HW, hind base width; FL, forelimbs stride length; HL, hindlimbs stride length; OV, overlap between fore and hindlimbs). **B.** Whisker-boxplots that show descriptions of the values of the variables measured in footprint test in the two different conditions at the two different ages (WT 3∶3 month-old WT, n = 23; WT 6∶6 month-old WT, n = 16, KO 3∶3 month-old D2KO, n = 30 and KO 6∶6 month-old D2KO, n = 19). VC, variability coefficient. **C.** Forest plots show squared omega (unbiased effect size measure, ω^2^) of condition (WT *vs*. KO), age (3 months-old *vs.* 6 months-old) and interaction of condition with age for each footprint parameter. Mean ω^2^ is indicated by a black square. 95% CI of ω^2^ is shown by two lines at both sides of the square. ω^2^ value is considered statistically significant when the lines do not touch the vertical axis in 0. Parameters are indicated on the left and ordered according parameter hierarchical clustering in WT mice (a dendrogram showing the result of that hierarchical clustering is on the left). Notice how condition effect is important in several gait variables (hind base width –HW–, forelimb stride length –FL– and hindlimb stride length –HL– and speed are affected). However, age shows only a mild non-significant tendency in gait speed and in variability of forelimb stride length. There is not statistically significant effect of interaction between condition and age and confidence intervals indicate that the possible effect of that interaction is probably very weak.

To evaluate the mice’s gait we used the footprint test [24] with minor modifications. Non-toxic waterproof paint was used to dip the mouse’s paws (red color was used for hindpaws and black for forepaws). The animals were required to run along a straight narrow tunnel (20 cm×20 cm×70 cm) with a 40 cm long sheet of white paper on the floor to record the prints. A dark goal box was positioned at the end of the tunnel to encourage the mouse to run towards a dark and safe environment. Footprints at the start and the end of the tunnel were excluded from the analysis as they correspond to the initiation and termination of the movement. Measurements for three-step cycles were averaged, considering a cycle as the distance from one pair of hind prints to the next. The parameters registered were the front base width, hind base width, the forelimb stride length, hind-limb stride length, the overlap between hind and forelimbs and the speed to walk the tunnel (Figure 1A).

#### Rotarod test

Motor coordination and balance were evaluated with the accelerating-rotarod test as described in Wei et al. [25] with some modifications. WT and D2KO mice were placed on an accelerating rotarod (Ugo Basile, Italy) that was programmed to accelerate from 4 to 44 rpm in 3 min and then hold at constant speed for further 2 min. The latency of the mice to fall off the rod was recorded over a maximum observation period of 5 min. Animals were given a session consisting of 3 trials per day with a 20 min inter-trial interval and repeatedly tested for 5 consecutive days. Data from 3 trials were averaged.

#### Four limb hanging wire test

Muscle strength was evaluated using the hanging wire test [26] in which mice were placed on the top of a wire cage lid quadrant (14×14 cm). The lid was gently shaken three times to improve the grip of mice and inverted horizontally 50 cm above a surface with bedding. The latency to fall was recorded over a maximum period of 120 s.

#### Balance beam test

The ability of the mice to maintain balance was evaluated as described in Arqué et al. [27]. Mice were placed on the center of a horizontal wooden bar (0.9 cm wide×50 cm long) 40 cm above a surface with bedding. The latency to fall was recorded over a maximum period of 40 s and two trials were performed. The activity on the bar was rated as (0) if the mouse stayed on the center and (1) if the mouse moved out of the center of the bar.

#### Coat-hanger test

Prehensile reflex and traction capacity was evaluated as described in Arqué et al. [27] with minor modifications. Mice were placed upside-down in the center of a horizontally positioned wire. First, the prehensile reflex was evaluated by scoring the behavior on the bar as it follows: (0) if the mice fell off the wire and (1) if the mice were able to hang for a 5 s period. During this 5 s period the traction capacity was evaluated by rating as (0) if the mice did not lift any of its limbs, (1) if it lifted one of the hind limbs and (2) if it lifted both of its hind limbs.

### Histological analyses

Nissl staining of CB sections (25 µm) was performed using toluidine blue in tissues from 6-month-old WT and D2KO mice as previously described [28].

Sections containing both gastrocnemius and soleus muscles (5 µm) from 6 month-old WT and D2KO mice were processed for hematoxylin (HHS32-1L; Sigma) eosin (HT110332-1L; Sigma) staining using routine protocols offering the possibility to study muscles with different predominance of slow-twitch and fast-twitch muscle fibers [29]. Furthermore, previous studies have detected a high expression of D2 in soleus muscle [12].

Histochemical analyses were made under bright field illumination using a Nikon Eclipse 80i microscope (×2, Numerical Aperture 0.06 and ×20, Numerical Aperture 0.50). Photomicrographs were acquired using a Nikon DS-Fi1 digital camera.

### Biochemical analyses

#### Hormonal determinations

Total T3 and T4 concentrations were determined by specific and highly sensitive radioimmunoassays in different brain regions, plasma and quadriceps muscle after extraction and purification of tissue extracts [30]. High specific activity T4 and T3 labeled with ^125^I were synthesized as described [30]. Quadriceps muscle was chosen due to the high muscle volume necessary to perform these determinations. Three and 6-month-old WT and D2KO mice were used, except for the muscle determination that was performed only in 6-month-old samples. For tissue assays, samples from several animals were pooled (tissue from 3 animals for CB, 2 for CTX, 3–4 for muscle). Serum samples were analyzed individually.

#### Deiodinase activities

The D1 and D2 activities were assayed as previously described [31] in different brain regions homogenates (1∶30, wt/vol) using 0.32 M sucrose, 10 mM 4-(2-hydroxyethyl)piperazine-1-ethanesulfonic acid (HEPES), and 10 mM dithiothreitol (DTT). In addition, quadriceps muscle homogenates were subjected to ultracentrifugation (100,000 g, 30 min) to obtain the microsomal fraction, which was also assayed. Before each assay, ^125^I-rT3 was purified from the iodide traces. D3 deiodination was blocked by adding 1 µM T3 in the reaction mix. Blanks were set up with homogenization buffer. The amount of iodide in the blanks was around 1% of the total radioactivity. Total deiodinase activity was assayed using ^125^I-labeled rT3, 2 nM rT3, and 20 mM DTT for 1 h at 37C and D2 activity by adding 1 mM propylthiouracil to the reaction mix [32]. D1 activity was determined by subtracting D2 activity from total deiodinase activity. Results are expressed in femtomols per hour per milligram of protein. Detection limits were 2 to 3 fmols/h • mg of protein.

### Data analyses

To analyze the importance of **condition and age in footprint variables,** squared omega (ω^2^, an unbiased estimate of proportion of variance associated with the grouping variable), was calculated in each footprint variable for condition (WT *vs.* D2KO), age (3 months *vs.* 6 months) and the interaction of condition and age. 95% confidence intervals (CI) were calculated by bias-corrected and accelerated bootstrap (1000 repetitions). Results were shown by Forest plot.

A stratified analysis by age was done for **rotarod** results. Two (one for 3-month-old animals and one for 6-month-old ones) general linear models were trained using “latency to fall” as the dependent variable and condition (WT *vs.* D2KO) and day (first day *vs.* second, third, fourth and fifth) as independent variables. Effect was assessed by beta coefficients and their 95% CI.

Random forests were trained as pattern recognition approaches. Three random forests were trained using condition as output and age with three different sets of data as inputs. The first set of data is formed by **footprint variables**. The latency to fall in the 5 different days of **rotarod** testing forms the second set of data. The third one groups the first and the second together. Notice that every mouse in the rotarod have footprint analysis, but only some with footprint analysis performed also in the rotarod. Accuracy of each random forest was measured as area under the ROC curve (AUC) and importance of each input variable was evaluated by total decrease in node impurities. A value for AUC>0.5 indicates a non-random effect and the nearer the value of AUC is to 1, the better one model is. On contrary to other analyses made, pattern recognition approaches allow us to study motor domains (gait or coordination measured by rotarod) as a whole and not only using isolated variables.

A Cox proportional hazards model was used to study the results of the **four limb hanging wire.** Latency to fall was used as dependent variable and condition (D2KO *vs*. WT, the later as reference), age (6 months *vs.* 3 months, the later as reference) and baseline hazard function and robust estimation of variance were used. Beta coefficients of each independent variable and its 95% CI were used in the result interpretation. Four Kaplan-Meier curves were drawn to assist in the interpretation of these results.

In the **balance beam test**, log-rank test was used to analyze differences between WT and D2KO in latencies to fall in the first and the second day and Fisher’s exact test was used to evaluate differences in activity on the bar. Prehensile reflex in the **coat-hanger test** was also analyzed with Fisher’s Exact Test.

To analyze differences in **TH levels** and **activities of deoidinases** between WT and D2KO mice, two-tailed unpaired Student’s t-tests were measured in different experiments for each age. We did not perform a two-way ANOVA of these data because experiments at different ages were independent.

The statistical analyses were performed using SPSS Statistics 19 and R software.

## Results

### 1. First-line evaluation of motor tasks

To test whether D2KO mice present gross motor abnormalities we decided to perform a series of commonly used tests that evaluate complex motor tasks as the animal’s gait, its coordination and balance and the muscle strength using the footprint test, the rotarod test and the four limb hanging wire test, respectively. As age can be an influential factor in these tests, we decided to study these performances at 3 and 6 months of age.

#### Footprint is altered in D2KO mice

D2KO mice walked slower (ω^2^∶0.530, 95% CI: 0.390 to 0.627), with shorter forelimb (ω^2^∶0.234, 95% CI: 0.082 to 0.389) and hindlimb stride (ω^2^∶0.212, 95% CI: 0.065 to 0.371) and with a hindlimb wider base of support (ω^2^∶0.243, 95% CI: 0.095 to 0.384) than WT mice (Figure 1B and C). Effect of age was not significant in footprint variables (Figure 1B). Differences between D2KO and WT remained stable between the 2 selected ages as no important effect of interaction between age and condition was detected (Figure 1B and C).

#### Rotarod performance is altered in D2KO mice

**Figure 2:**
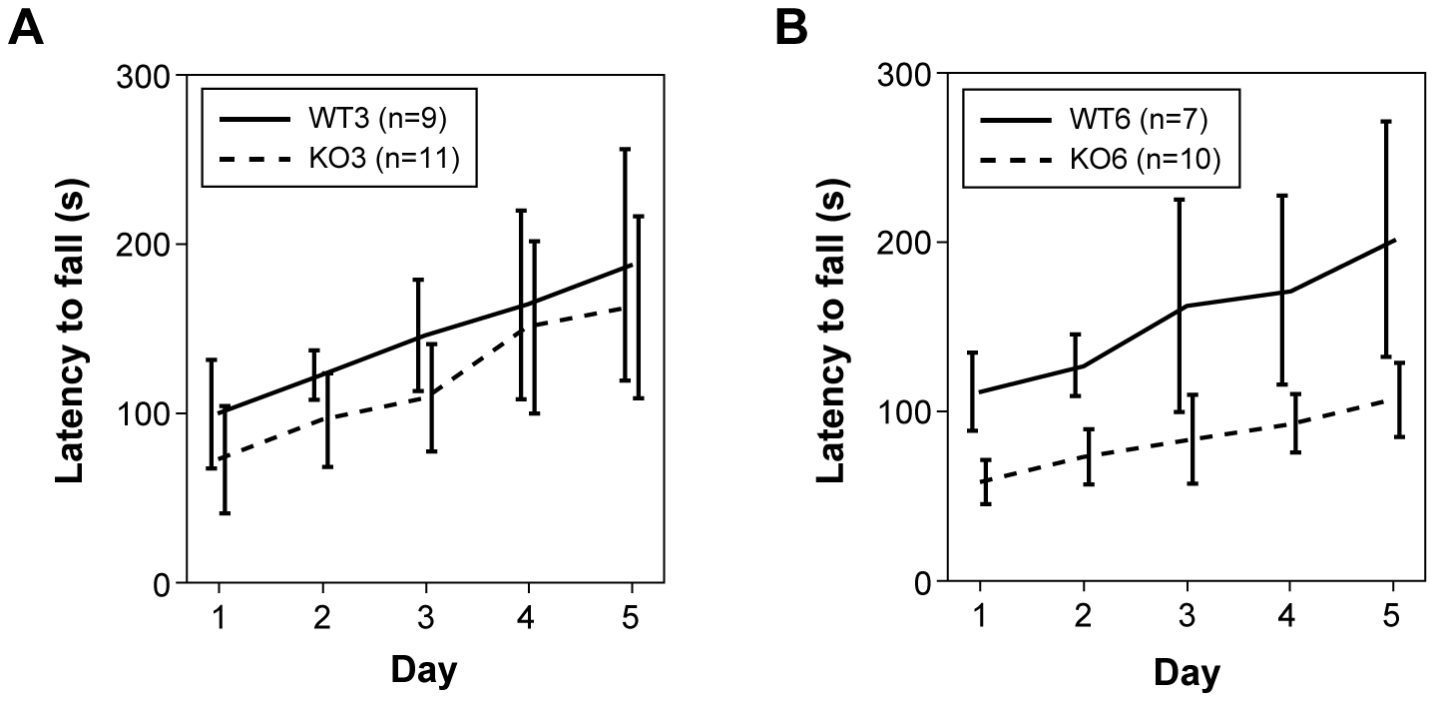
Latency to fall in rotarod test at 3-month-old (A) and 6-month-old (B) mice. Data for the 5 days of evaluation is shown. 95% CI is represented with error bars. Notice how only small non-significant tendency to differ exist between genotypes at 3 months. Clearly, 6-month-old D2KO mice show a significantly lower latency than WT mice.

At 3 months of age, latency to fall in the rotarod test progressively increased from the first day both in WT and D2KO mice. No statistically significant effect was proved for condition although a slight tendency to decrease in D2KO mice was detected (*P* = 0.192). At 6 months of age, falling latency also increased with days both in WT and D2KO mice, but in this case, D2KO mice fell significantly earlier than WT mice. On average, D2KO fell from 38 to 105 seconds earlier than WT mice (*P*<0.001; Figure 2).

#### Footprint variables and rotarod can discriminate between D2KO mice and WT mice

**Figure 3:**
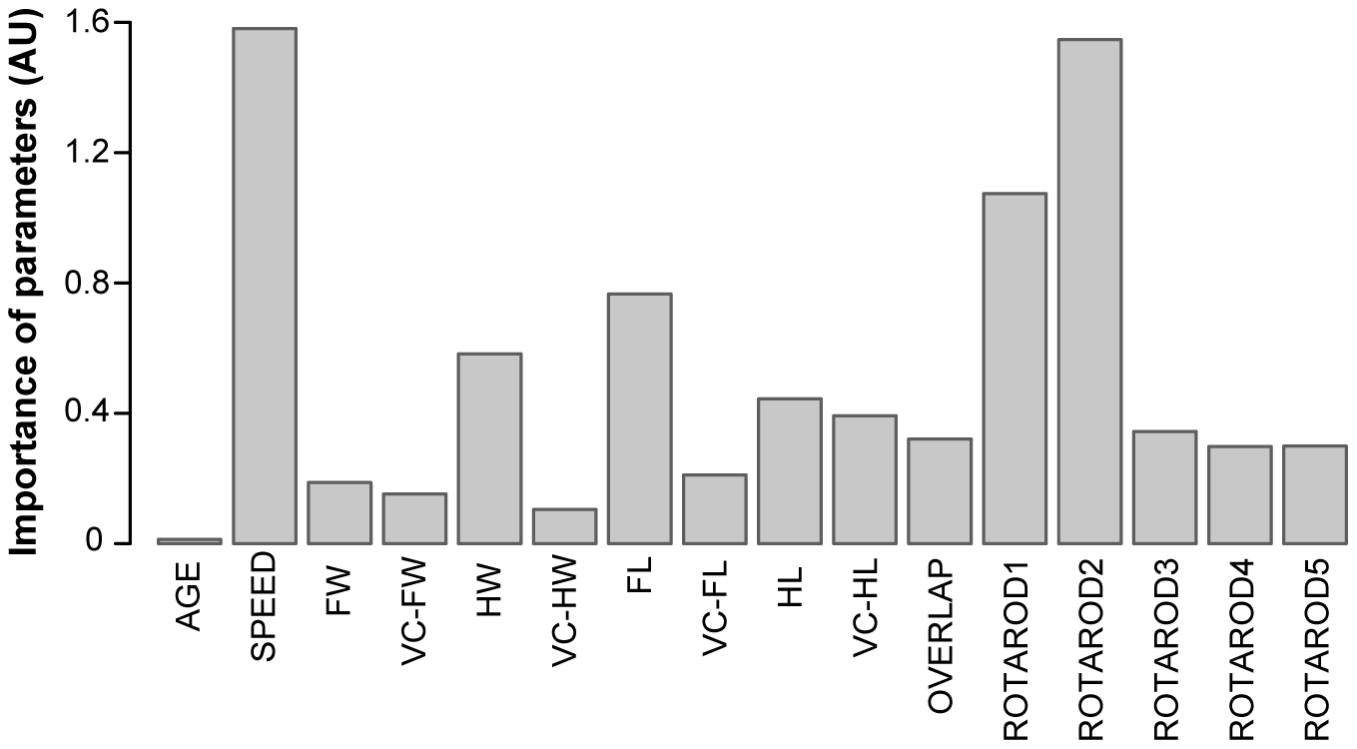
Importance of footprint and rotarod parameters for the prediction of animal condition. The barplot represents the importance of footprint and rotarod parameters in the prediction of animal condition in the random forest trained with those parameters (AU, arbitrary units). ROTAROD1 to 5 indicate the latency to fall in the 1^st^ to 5^th^ day of testing. For meaning of the rest of abbreviations, see Figure 1. The higher the bar is, the more important a particular variable is in the prediction. The most important variable is speed. Other relevant variables are latency to fall in the first and second rotarod days –ROTAROD1,2–, forelimb stride length –FL– and hindlimb stride width –HW–.

Pattern recognition analysis of the data obtained from footprint studies revealed a particular way of walking of D2KO mice. Random forest trained with footprint variables showed higher discrimination performance (AUC = 0.909) than that trained with rotarod data (AUC = 0.795; Figure S1). Taken data together as input, AUC was 0.914, nearly the same as footprint variable alone (Figure S1). This indicates an alteration in global gait pattern. The most important variables in distinguishing D2KO from WT animals were walking speed, forelimb stride length, and hind base width for footprint measures, and the first and the second day of the rotarod test (Figure 3 and S2).

#### Muscle strength is decreased in D2KO mice

**Figure 4:**
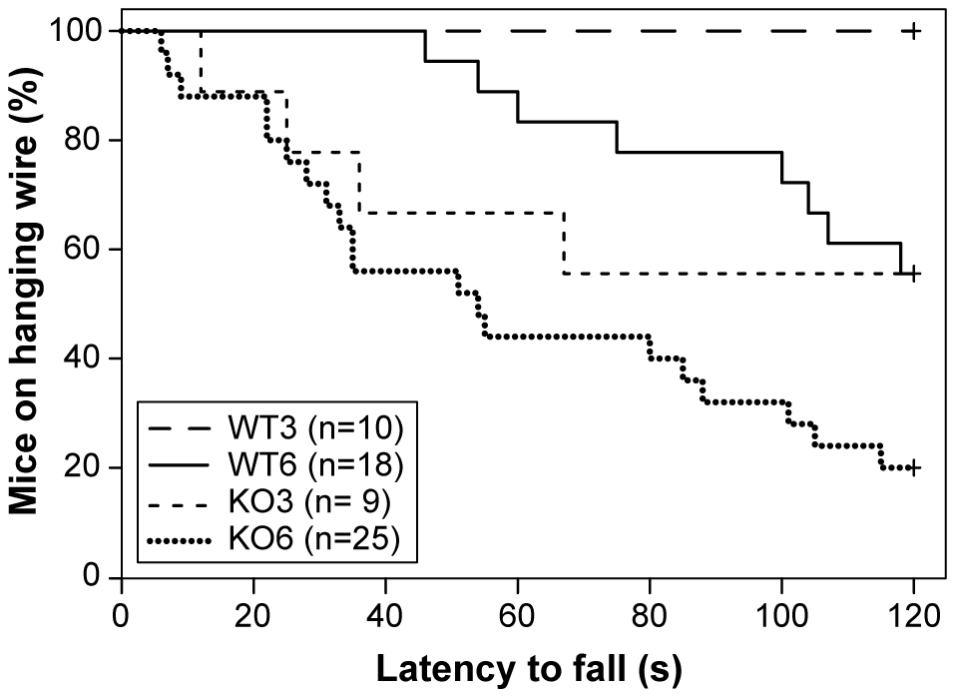
Alteration in latency of fall in four limb hanging wire test. Kaplan-Meier plot showing latency to fall in four limb hanging wire test. The horizontal axis shows time to fall in seconds. Notice that experiment is stopped at 120 seconds. In vertical axis, percentage of mice that have not fallen is represented. A line shows the evolution in each group. WT mice at 3 months did not fall during the observation period, but more than a 40% of 6-month-old WT mice had fallen at 120 seconds. KO mice showed a lower latency to fall at both ages.

Four limb hanging wire test evidenced a reduction of muscle strength in D2KO mice at both ages in comparison to matched age control mice (Figure 4). Multivariate survival analysis showed significant effects for condition, age and interaction of age with condition. D2KO mice fell earlier than WT (beta coefficient for D2KO condition compared with WT: 19.3; 95% CI 18.1 to 20.5; *P*<0.001). Older mice also fell earlier than younger ones (beta coefficient for 6-month-old mice, compared with 3-month-old-mice: 19; 95% CI 18.1 to 19.9; *P*<0.001), but this effect of age is less marked in D2KO group as demonstrated by the negative beta coefficient for interaction (beta coefficient: −18.2; 95% CI −19.7 to −17.7; *P*<0.001) and by the survival curves (Figure 4).

### 2. Second-line evaluation of motor tasks

As disorders were observed in gait, coordination and muscle strength, a more comprehensive evaluation was done. We decided to further characterize D2KO mice’s phenotype by studying 6-month-old animal’s equilibrium, prehensile reflex and traction capacity.

#### Equilibrium is not affected in D2KO mice

**Figure 5:**
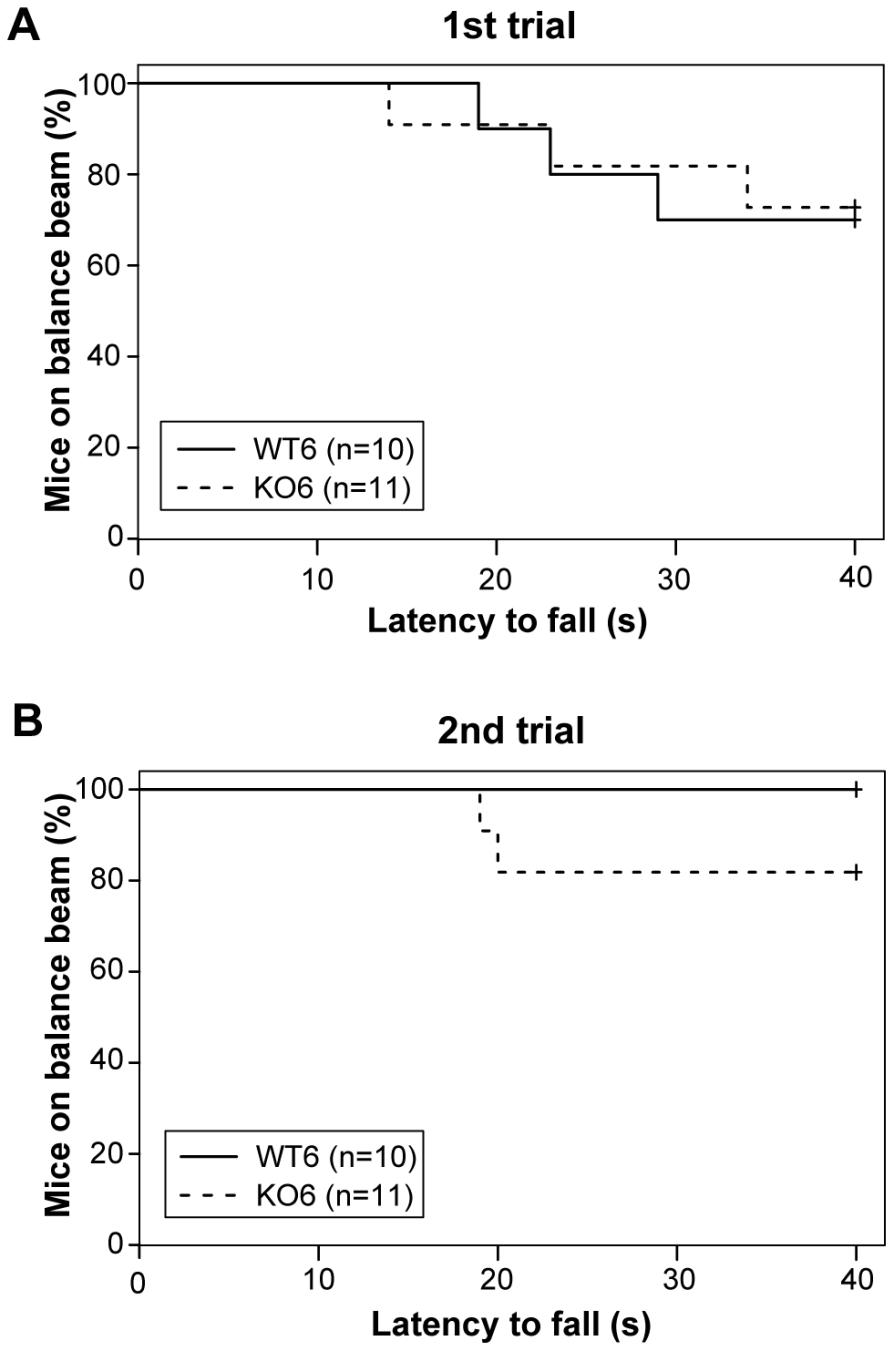
No differences in balance beam test. Kaplan-Meier curves that show latency to fall in balance beam test at first (**A**) and second (**B**) trial in WT (plain line) and D2KO mice (dotted line). No significant differences were detected in the log-rank test at both trials. However, none of WT mice but some D2KO fall during the second trial.

Balance beam test was not altered in the D2KO mice (Figure 5). Latency to fall differed neither in the first trial (χ^2^<0.01; *P* = 0.889) nor in the second (χ^2^ = 1.9; *P* = 0.167). Furthermore, no significant differences regarding the activity on the bar could be observed among these groups (30.0% of the WT mice and 27.3% of the D2KO mice moved out the center of the bar; *P* = 1.000).

#### Prehensile reflex is affected in D2KO mice

Differences between genotypes were found in the coat-hanger test as D2KO mice showed a severe impairment on their prehensile reflex. While 92.9% of the WT mice were able to remain hanging from the wire for 5 s, only 33.3% of the D2KO mice were able to perform this task (n_WT_ = 14, n_KO_ = 24; *P* = 0.001).

#### Evaluation of traction capacity

Traction capacity was evaluated in the coat-hanger test during the 5 first seconds in which the mouse is hanging from the bar. As the vast majority of the D2KO mice were not able to remain hanging for that long, it was impossible to evaluate traction capacity in those animals. However, from the few D2KO mice that remained hanging, there seems to be a tendency to a decreased traction capacity in comparison to WT mice. Unfortunately, due to the low number of D2KO mice that remain hanging for 5 seconds a statistical analysis could not be performed.

### 3. Histological analyses

To determine if the abnormalities observed during these tasks were due to severe and gross defects in the structure of the CB and/or muscle fibers, these two tissues were histologically studied in 6-month-old animals.

#### D2KO mice do not present gross cerebellar structural abnormalities

**Figure 6:**
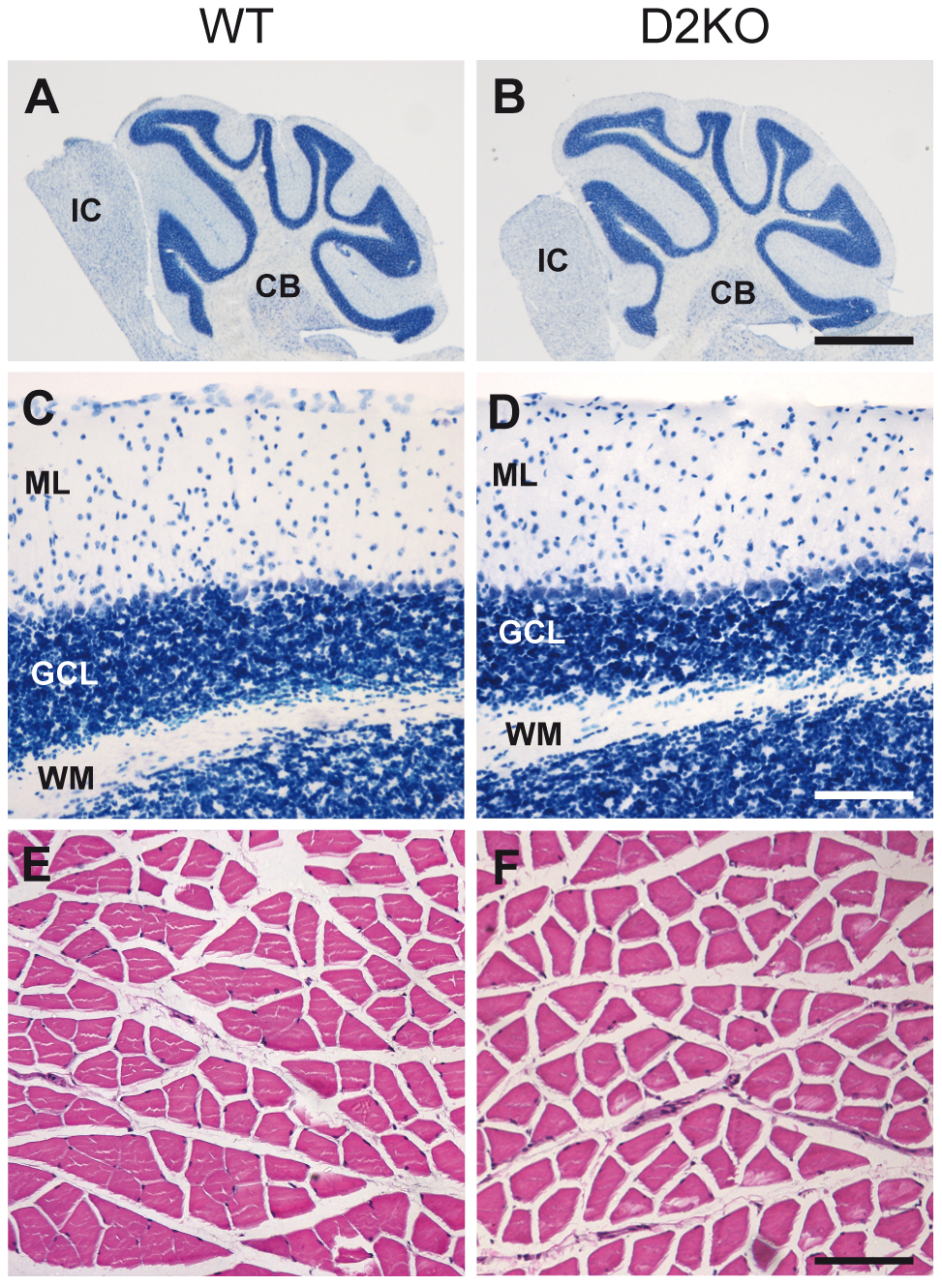
Histological studies in the cerebellum and skeletal muscle. Microphotographs showing representative images of Nissl-stained tissue sections from cerebellum (**A–D**) and hematoxylin-eosin stained tissue sections from gastrocnemius skeletal muscle (**E,F**) in 6-month-old WT (**A, C, E**) and D2KO (**B, D, F**) mice**. A, B** Low magnification images showing a complete cerebellum in a tissue section. **C, D** Detail of cerebellar layers. Gross structural alterations were found neither in cerebellum (**A–D**) nor in skeletal muscle (**E,F**). Tissues from 3 animals of each experimental condition were used in these studies. CB: cerebellum, ML: Molecular layer of the cerebellum, GCL: granule cell layer of the cerebellum, WM: white matter, IF: inferior colliculus. Scale bars, 1 mm for A–B and 100 µm for C–F.

It has been well established that cerebellar development is a classic TH dependent process [33]. As alterations were observed in the rotarod performance of D2KO mice, we decided to search for abnormalities in the histology of the cerebellar cortex. However, no gross structural abnormalities were detected in the Nissl-stained sections from the D2KO CB that showed a normal cytoarchitecture of the molecular, Purkinje and granule cell layers of the cerebellar cortex (Figure 6 A–D).

#### Skeletal muscle structure is not altered in D2KO mice

We did not find in hematoxylin-eosin stained tissue sections from gastrocnemius and soleus of D2KO mice any signs of atrophy, dystrophy or muscular degeneration such as necrosis, weaker stained fibers, alterations in the number and location of nuclei, higher vascularization, rounded shaped fibers, presence of lymphocytes and eosinophils inclusions or vacuolization (Figure 6 E–F).

### 4. Thyroidal status

To better understand these neurological alterations we also analyzed the thyroidal status of several brain regions and quadriceps muscle by monitoring TH levels and deiodinase activities.

#### TH levels are altered in several tissues in D2KO mice

The analyses of T4 and T3 plasma levels of WT mice were consistent with previous measurements in euthyroid mice of the same age [28]. Plasma T4 levels were significantly higher in D2KO mice (Table 2), both at 3 and 6 months of age and T3 levels did not show significant differences between WT and D2KO mice.

**Table 2:** Thyroid hormones levels in plasma and tissues.

Hormone	Age	Genotype	Plasma(ng/ml)	Cerebellum(ng/g)	Cortex(ng/g)	Skeletal Muscle(ng/g)
T4	3 m	WT	20.56±2.15 (10)	5.54±0.32 (4)	3.83±0.22 (7)	–
		KO	37.69±3.56^b^ (14)	3.85±0.49^a^ (6)	5.52±0.33^b^ (4)	–
	6 m	WT	34.48±1.18 (10)	2.86±0.43 (7)	2.72±0.22 (11)	2.17±0.10 (11)
		KO	44.69±2.01^c^ (9)	2.40±0.15^NS^ (7)	3.55±0.22^a^ (9)	2.10±0.09^NS^ (10)
T3	3 m	WT	1.78±0.31 (9)	3.33±0.18 (4)	3.32±0.15 (6)	–
		KO	1.64±0.16^NS^ (14)	2.74±0.20^NS^ (5)	2.07±0.35^b^ (7)	–
	6 m	WT	0.68±0.04 (10)	2.41±0.27 (7)	1.06±0.09 (11)	0.43±0.03 (10)
		KO	0.79±0.05^NS^ (9)	1.87±0.12^NS^ (7)	0.76±0.05^b^ (9)	0.29±0.02^b^ (9)

Data are means ± SE. NS: not statistically significant. CB: cerebellum, CTX: cerebral cortex.

a *P*<0.05; ^b^ *P*<0.01; ^c^ *P*<0.001 *vs.* WT. The number of samples per group is stated in parenthesis.

In the brain, T4 and T3 levels were measured in CB and CTX (Table 2). In CB, T4 levels were reduced in D2KO mice in comparison to WT at 3 months and T3 levels were comparable between both groups. In CTX, T4 levels were significantly higher in D2KO mice at both ages, as it occurred in T4 plasma levels, but T3 levels were reduced in D2KO mice in comparison to WT both at 3 and 6 months of age.

In skeletal muscle T4 levels were similar between genotypes, but T3 levels were significantly reduced in D2KO mice (Table 2).

#### D2 and D1 activities

**Figure 7:**
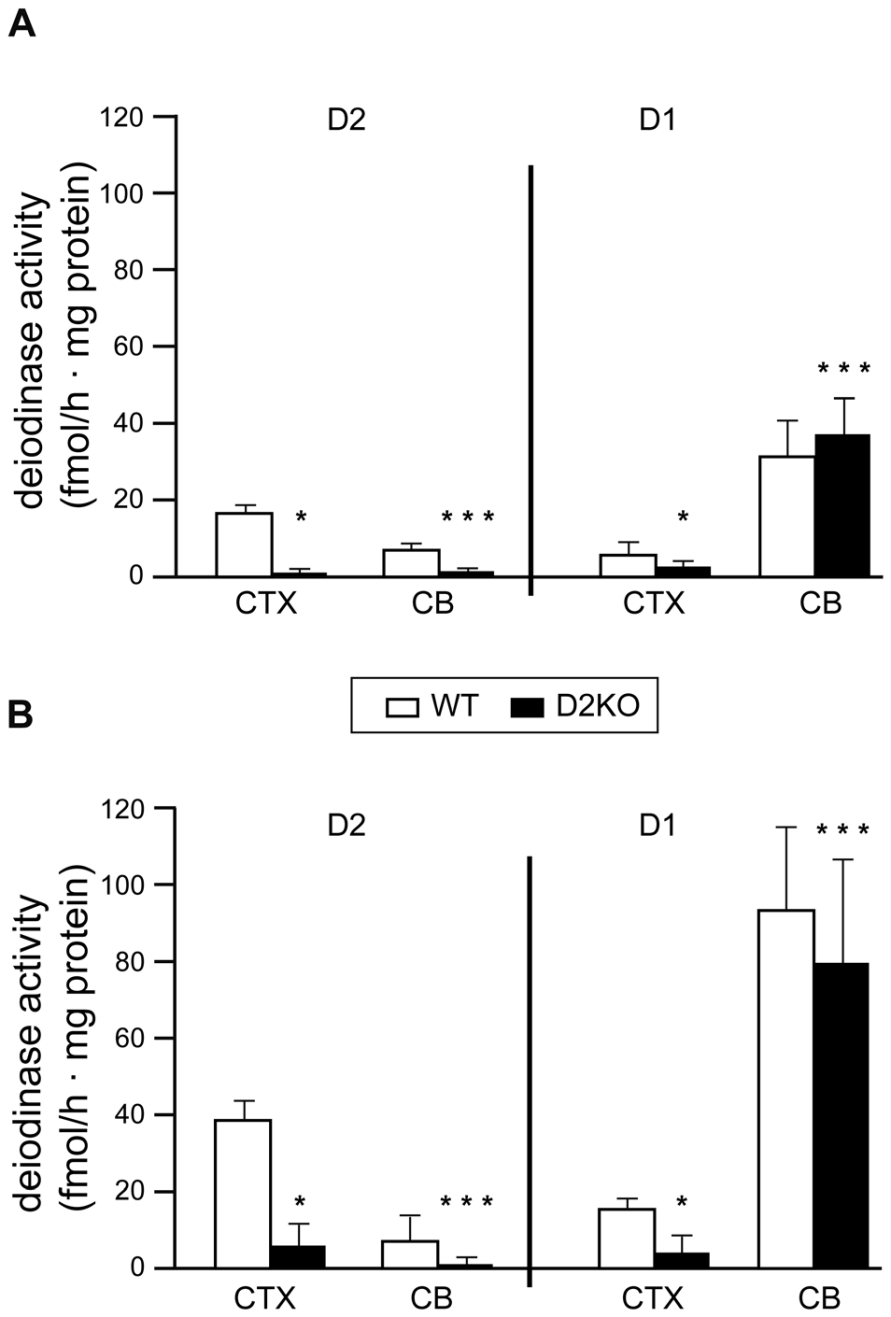
Differences in the activities of deoidinases. Barplots represent D2 and D1 activities in cerebral cortex (CTX) and cerebellum (CB) of 3-months-old (**A**) and 6-months-old (**B**) WT and D2KO mice. Although statistical significant difference exists in D1 activity in the cortex between WT and D2KO at 6 months, this difference is not considered as relevant because D1 activity is very low in both conditions. Tissues from 5 to 6 animals of each condition were used for these assays. ***P-value less than 0.001; **P-value less than 0.01.

Deiodinases activities were also assayed in the CB and CTX of 3- and 6-month-old WT and D2KO mice to determine the local generation of T3 in these brain regions (Figure 7).

In WT mice, D2 activity was clearly detected in the CTX and low levels, near the detection limit, were found in the CB (Figure 7). Negligible D2 activity was detected in D2KO mice.

D1 activity was very low in the CTX of WT and D2KO mice (Figure 7). Nevertheless, a high D1 activity was observed in the CB of both WT and D2KO mice, which was 6-fold the D1 activity in the CTX. No significant differences were observed in the D1 activity at the CB between WT and D2KO mice (Figure 7).

D1 and D2 activities were also measured in quadriceps muscle of 6-months-old WT and D2KO mice in microsomal fractions (n = 6 for each condition). D2 activity was very low in WT mice muscles, near the detection limits (4.1±2.8 fmols/h/mg protein). No D2 activity was detected in samples from D2KO mice. D1 activity was detected in microsomal extracts, and no significant differences were found between groups (D1 (WT) = 9.8±3.6 and D1 (KO) = 5.0±3.9 fmols/h/mg protein; *P* = 0.082).

## Discussion

In this study, remarkable abnormalities in relevant motor tasks in adult D2KO mice such as gait, coordination, muscle strength and prehensile reflex were found. These alterations could be likely related with a complex motor phenotype involving abnormalities at several levels of the motor system. These results could be explained by the important role of D2 in the homeostasis of TH at different areas of this system [6], [10], [34]. D2 is a key regulator of TH metabolism in the brain (around 80% of intracerebral T3 bound to nuclear receptors is derived from circulating T4 by local D2 activity [35], [36]) and in the skeletal muscle, where it is crucial for normal development and repair [10].

### A severe motor impairment appears in adulthood in D2KO mice

We have found that gait and coordination are significantly altered in D2KO mice. Our results showed that mice with D2 deficiency can be well discriminated from WT in the basis of data coming from footprint and rotarod tests. The pattern detected in D2KO mice (slower walking, shorter steps, increased hindlimb base of support and coordination impairment) could be the consequence of several alterations in the motor system. Similar gait changes have been described in premature ageing or in ataxic disorders [37]. However, there is limited data in the literature from footprint for other types of lesions in the motor system. We also found a reduction in muscle strength (evidenced by a decreased hanging latency in the hanging wire and prehensile reflex tests) in D2KO mice that could be the underlying consequence of myopathy, corticospinal alterations or both. All these results could indicate that the abnormal motor phenotype is actually a complex one in which several areas of the motor system could be involved.

Previous motor evaluations of these mice at younger ages did not find clear neurological disturbances [23]. This was an unexpected result given the supposed key role of D2 in brain TH homeostasis, especially in early CNS development [6], [20]. In contrast to the practically normal phenotype at young ages, the motor phenotype in adult D2KO mice is severely impaired. For instance, the rotarod test evidenced that the older the mice were, the worse performance they tended to exhibit in comparison to WT ones. This points to a relevant role of age in the appearance of alterations secondary to D2 deficiency. Progressive failure of compensating mechanisms or disruption of normal ageing processes by the lack of D2 could explain the late-onset appearance of the abnormal phenotype in adult stages. Moreover, some compensating mechanisms could even be brain region- and age-dependent. For example, adult D2KO mice showed nearly normal T3 levels in the CB (in contrast to a 50% reduction in 2 week-old mice), probably due to the constitutively high D1 activity in this region at this age, which provides an additional way to generate local T3. In contrast, in the cortex where D1 activity at this age is very low, T3 levels cannot be compensated in D2KO mice therefore exhibiting a 40% reduction in comparison to WT mice. Although D1 has not been considered to have an important role in brain, previous work have described that D1 is found in brain [8], [38] and is regulated by hypothyroidism at least in the areas and ages reported [38]. The low T3 in the cortex could be underlying impairments at the corticospinal tract that can lead to the observed weakness. Due to the alterations in gait and rotarod execution in D2KO mice and because of the important role of TH in cerebellar development, we decided to study cerebellar histology, but no apparent alterations in Nissl-stained sections were detected. The lack of gross abnormalities in this region could be explained by the nearly euthyroid state probably maintained by D1 activity as explained above.

Another explanation for this phenotype could be the low content of T3 in muscle in D2KO mice. This is the first study that directly measures the TH levels in adult skeletal muscle detecting low tissue T3 levels in D2KO mice despite normal T3 serum levels. This result is in agreement with previous studies in which gene expression analysis both *in vitro* [39] and *in vivo* [11] showed a decreased expression of T3-target genes pattern in muscle regardless of normal serum T3 concentration. Despite the low levels of T3, D2 activity measurements showed a very low activity in WT mice and no activity in D2KO mice. It is well accepted that D2 activity determinations in muscle are technically difficult, so higher activities should not be discarded. Furthermore, as skeletal muscle comprises 40–50% of body mass, even low D2 activity could have a significant effect on muscle metabolic rate [12]. As we have reported significant lower T3 levels in muscle, it looks likely that D2 contributes significantly to muscle T3 despite its low activity. Moreover, D2 activity might play a more relevant role in response to tissue demands (such as growth, repair or others) as it is involved in muscle myogenesis and regeneration [11].

Skeletal muscle histology was also studied because of D2KO mice weakness and the involvement of D2 in muscle physiology [10]–[12]. Although no gross histological abnormalities in hematoxylin eosin sections of gastrocnemius and soleus skeletal muscles could be found in D2KO mice, further histological, metabolic and neurophysiologic studies will be needed to better clarify the origin of weakness in these mice.

From these results it is difficult to discriminate if the defects in motor tasks are due to central nervous system impairment or a skeletal muscle dysfunction or it could even be both. What it seems clear is that D2 deficiency causes a more severe impairment than traditionally thought and highlights the need of T3 availability in certain tissues (such as CNS and/or muscle) at certain stages provided by D2.

Further studies will be needed to determine if motor alterations in D2KO mice are irreversible. This study provides a methodology to analyze this, with a selection of important altered parameters to monitor the response to possible treatments with thyroid hormones or thyroid hormones analogues.

### Implications for human health

Based on our findings in D2KO mice, mutations in *DIO2* in humans should be associated with motor manifestations. There are several reasons why a *DIO2* has not been related with neurological disorders: namely, the lack of expected clinical features related to the classical presentation of TH congenital disorders in D2KO mice [40], the lack of expected clinical laboratory profile of hypothyroidism [18] and the unexpected late-onset of clinical symptoms that appear only to become evident at adult stages in mice. With the data available, it is very difficult to predict plasma TSH, T3 and T4 levels in humans with mutations in *DIO2* [41]–[43]. A significant part of patients exhibiting motor disorders are still to be etiologically diagnosed and the description of D2 involvement in motor system could indicate a role in motor disorders of genes related with the action of thyroid hormones.

Based on previous studies in mice, it is tempting to speculate that the potential patients with mutations in *DIO2* could suffer from deafness, thermogenesis alterations, metabolic syndrome and skeletal abnormalities [11], [19]–[21], [44], [45]. Here, we present additional motor signs. If the D2 deficient mouse phenotype is similar to human diseases, patients could have impairment in gait and coordination with weakness.

## Supporting Information

Figure S1 **Prediction of animal condition by footprint and rotarod parameters.** ROC curves that represent the goodness of fit of three random forests in the prediction of condition (WT or D2KO). **A.** Random forest trained only with footprint variables, **B.** with rotarod variables, and **C.** with both. The best performance, higher AUC, is the one of the random forest trained with footprint and rotarod parameters (**C**). However, ROC curves show in panels **A** and **C** are quite similar.
(TIF)

Figure S2 **Importance of footprint or rotarod parameters for the prediction of animal condition. A.** Bar plot that represents the importance of footprint parameters in the prediction of animal condition in the random forest trained with those parameters. **B.** Bar plot that represents the importance of rotarod parameters in the prediction of animal condition in the random forest trained with those parameters. The higher the bar is, the more important a particular variable is in the prediction.
(TIF)
