# Supplementary figures and images for: Abnormal Motor Phenotype at Adult Stages in Mice Lacking Type 2 Deiodinase
Source: PLoS One. 2014 Aug 1;9(8):e103857. doi: 10.1371/journal.pone.0103857 (PMC4118963; doi:10.1371/journal.pone.0103857)

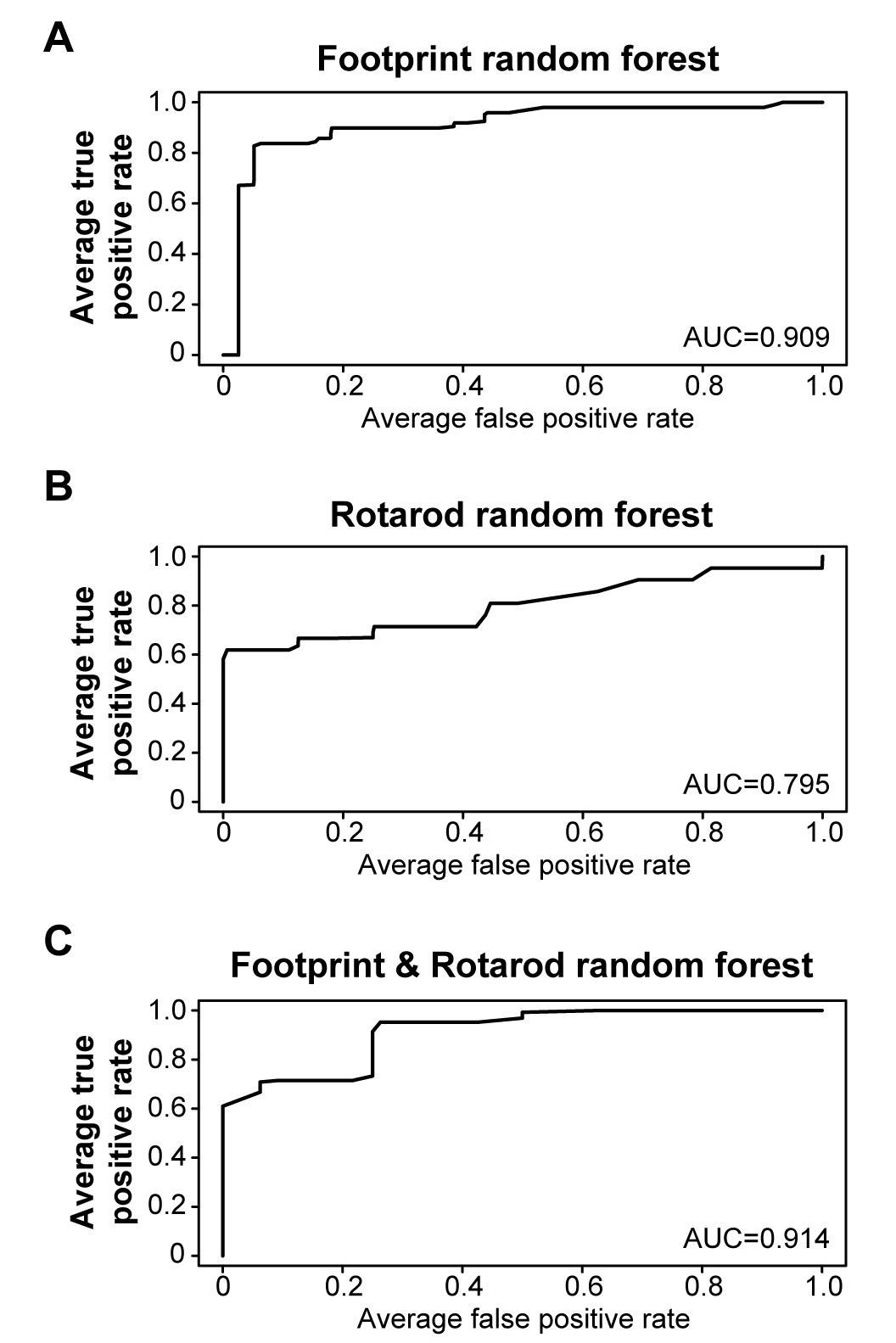

Supplement: Figure S1 — Prediction of animal condition by footprint and rotarod parameters. ROC curves that represent the goodness of fit of three random forests in the prediction of condition (WT or D2KO). A. Random forest trained only with footprint variables, B. with rotarod variables, and C. with both. The best performance, higher AUC, is the one of the random forest trained with footprint and rotarod parameters (C). However, ROC curves show in panels A and C are quite similar. (TIF) [file pone.0103857.s001.tif]

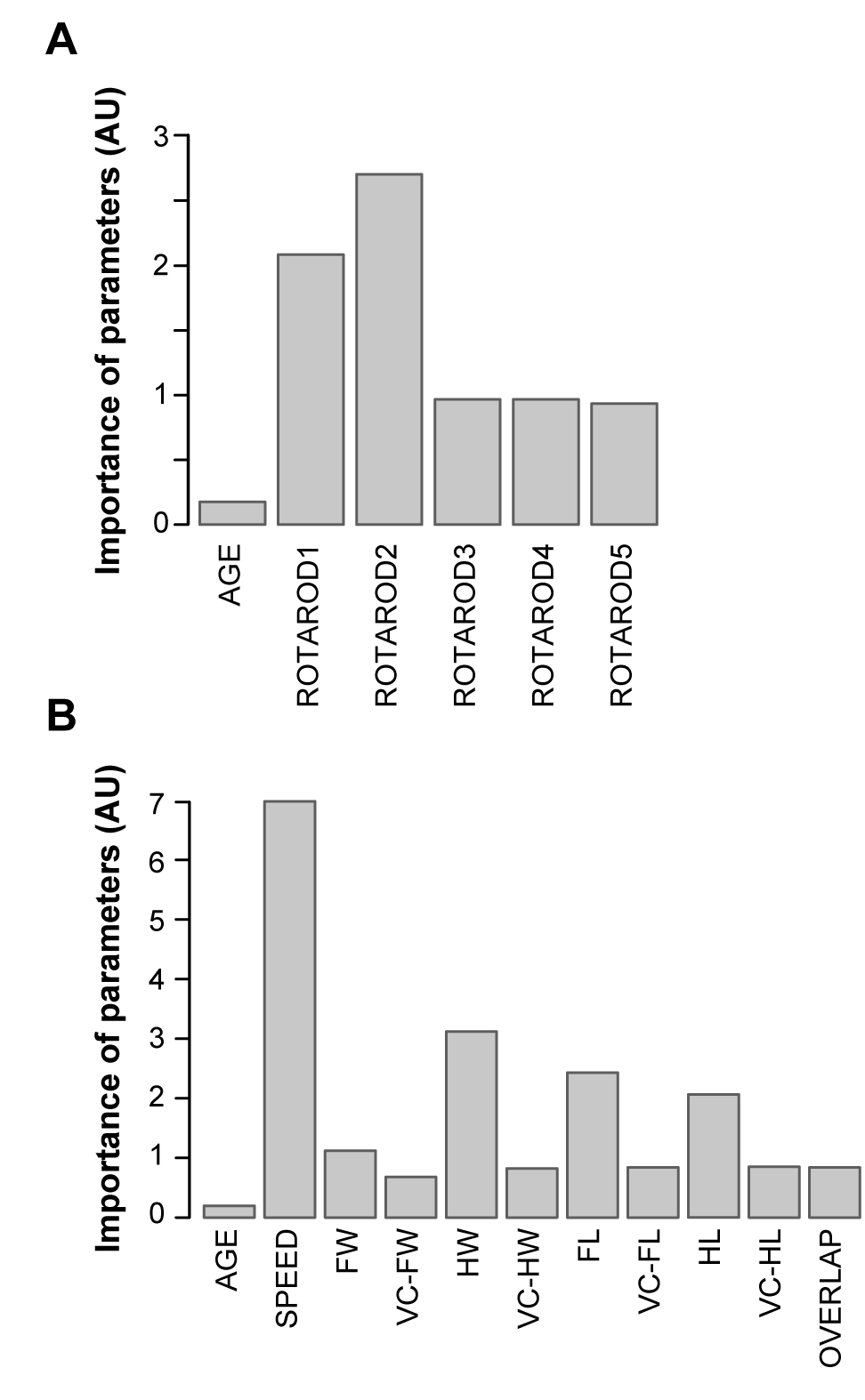

Supplement: Figure S2 — Importance of footprint or rotarod parameters for the prediction of animal condition. A. Bar plot that represents the importance of footprint parameters in the prediction of animal condition in the random forest trained with those parameters. B. Bar plot that represents the importance of rotarod parameters in the prediction of animal condition in the random forest trained with those parameters. The higher the bar is, the more important a particular variable is in the prediction. (TIF) [file pone.0103857.s002.tif]
